# Low‐Voltage Type 1 ECG Is Associated With Fatal Ventricular Tachyarrhythmia in Brugada Syndrome

**DOI:** 10.1161/JAHA.118.009713

**Published:** 2018-11-02

**Authors:** Satoshi Nagase, Tsukasa Kamakura, Naoya Kataoka, Mitsuru Wada, Kenichiro Yamagata, Kohei Ishibashi, Yuko Y. Inoue, Koji Miyamoto, Takashi Noda, Takeshi Aiba, Chisato Izumi, Teruo Noguchi, Satoshi Yasuda, Wataru Shimizu, Shiro Kamakura, Kengo Kusano

**Affiliations:** ^1^ Division of Arrhythmia and Electrophysiology Department of Cardiovascular Medicine National Cerebral and Cardiovascular Center Suita Japan; ^2^ Department of Cardiovascular Medicine Nippon Medical School Tokyo Japan

**Keywords:** Brugada syndrome, electrocardiography, sudden cardiac death, ventricular fibrillation, Sudden Cardiac Death, Ventricular Fibrillation

## Abstract

**Background:**

Epicardial mapping can reveal low‐voltage areas on the right ventricular outflow tract in patients with Brugada syndrome with several ventricular fibrillation (VF) episodes. A type 1 ECG is associated with an abnormal electrogram on right ventricular outflow tract epicardium. This study investigated the clinical significance of the amplitude of type 1 ECGs in patients with Brugada syndrome.

**Methods and Results:**

In 209 patients with Brugada syndrome with a spontaneous type 1 ECG (26 resuscitated from VF, 54 with syncope, and 129 asymptomatic), the amplitude of the ECG in leads exhibiting type 1 was measured among V1 to V3 leads positioned in the standard and upper 1 and 2 intercostal spaces. The number of ECG leads exhibiting type 1 did not differ among groups. The averaged amplitude of type 1 ECG was, however, significantly smaller in the group resuscitated from VF than in the asymptomatic group (*P*<0.05). Moreover, the minimum amplitude of type 1 ECG was significantly smaller in the group resuscitated from VF than in the group with syncope and the asymptomatic group (*P*<0.05 and *P*<0.01, respectively). During follow‐up (56±48 months), VF occurred in 29 patients. Kaplan‐Meier analysis revealed that patients with the minimum amplitude of type 1 ECG lower than or at the median value had a higher incidence of VF (log‐rank test, *P*<0.01). In multivariate analysis, syncope, past VF episode, and minimum amplitude of type 1 ECG ≤0.8 mV were independent predictors of VF events during follow‐up.

**Conclusions:**

Low‐voltage type 1 ECG is highly and independently related to fatal ventricular tachyarrhythmia in patients with Brugada syndrome.

**Clinical Perspective**

**What Is New?**

- This was the first study evaluating the association between the amplitude of a type 1 ECG and fatal arrhythmic events in Brugada syndrome.

**What Are the Clinical Implications?**

- Low‐voltage type 1 ECG among the right precordial leads on the standard and upper intercostal spaces highly and independently correlates with ventricular tachyarrhythmia in patients with Brugada syndrome.

Brugada syndrome (BrS) is characterized by ST‐segment elevation in the right precordial leads and an increased risk of ventricular fibrillation (VF).^1^ Risk stratification in BrS is an important consideration, and several predictive factors for future VF events have been reported.^2^, ^3^, ^4^, ^5^, ^6^, ^7^, ^8^, ^9^, ^10^, ^11^, ^12^, ^13^, ^14^, ^15^, ^16^, ^17^, ^18^, ^19^

Recording a type 1 ECG among the right precordial leads positioned on the standard or upper intercostal space (ICS) is essential for the diagnosis of BrS.^20^ Several reports suggested that the spontaneous appearance of a type 1 ECG is strongly related to the occurrence of VF.^3^, ^10^, ^11^, ^21^ Anatomical investigations revealed that the ECG lead exhibiting type 1 morphological features correlates with the location of the right ventricular outflow tract (RVOT).^22^, ^23^ Recently, direct epicardial mapping using the subxiphoid approach has revealed low‐voltage areas with fragmented and delayed potential on the RVOT in patients with BrS with several VF episodes.^24^, ^25^, ^26^, ^27^ Moreover, catheter ablation on these low‐voltage areas with fractionated potential suppressed the occurrence of VF, with disappearance of the type 1 ECG in the right precordial leads that are anatomically close to the RVOT. Thus, a type 1 ECG can be associated with the low‐voltage area on the RVOT in patients with BrS.

The aim of this study is to investigate the clinical significance of the amplitude of a type 1 ECG on the right precordial leads in patients with BrS.

## Methods

The data, analytic methods, and study materials will not be made available to other researchers for purposes of reproducing the results or replicating the procedure.

### Patient Population and Clinical Data

Among 352 patients who had been given the diagnosis of BrS at the National Cerebral and Cardiovascular Center, we retrospectively enrolled 209 consecutively in whom a spontaneous type 1 ECG was detected and right precordial leads on the upper ICS were recorded. All patients were diagnosed as having BrS on the basis of the Heart Rhythm Society, European Heart Rhythm Association, and Asia Pacific Heart Rhythm Society expert consensus statement after a positive spontaneous type 1 ECG recording in lead V1 or V2 on the standard or upper ICS.^20^ Patients with structural cardiac abnormalities on transthoracic echocardiography were excluded. Clinical data, including data on age, sex, family history of sudden cardiac death (for those <45 years of age), history of syncope episodes, history of VF episodes, and VF inducibility with programmed ventricular pacing, were obtained from patient records. Patients were divided into 3 groups according to baseline symptoms: a group with a previous VF episode (group VF), a group with a previous syncope episode (SY group), and a group without any symptoms (AS group). Follow‐up data defined the start of follow‐up as the first recording of a type 1 ECG and the end of follow‐up as a VF event or the last visit. This study was approved by the Institutional Research Board of the National Cerebral and Cardiovascular Center.

### ECG Analysis

**Figure 1:**
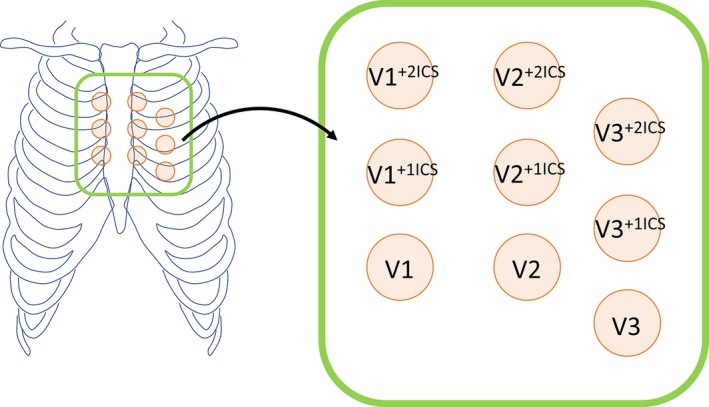
Position of 9 right precordial ECG leads analyzed in this study. In this study, V1 to V3 leads positioned in the standard and upper 1 and 2 intercostal spaces (+1ICS and +2ICS, respectively) were recorded for evaluation of the number and amplitude of type 1 ECG.

**Figure 2:**
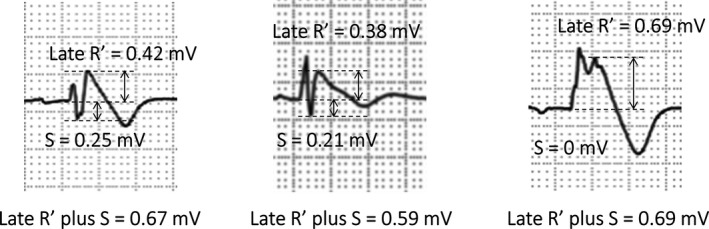
Measurements of ECG parameters in leads showing type 1. Among the 9 right precordial leads, the amplitudes of late R′, S, and late R′ plus S were evaluated in each ECG lead showing type 1.

In addition to a standard 12‐lead ECG, we evaluated ECG leads V1 to V3 on the upper 1 and 2 ICSs in all patients. Consequently, the 9 special right precordial leads V1 to V3 on the standard and upper 1 and 2 ICSs were evaluated in this study (Figure 1). The amplitude from the isoelectric line to the peak of the late R′ and to the nadir of the S waves in each ECG lead exhibiting type 1 with first documentation among the 9 right precordial leads was measured. Then, the averaged and minimum amplitudes of late R′, S, and the sum of late R′ plus S waves were analyzed (Figure 2). The number of ECG leads exhibiting type 1 was also evaluated among the 9 right precordial leads. An inferolateral early repolarization pattern was defined as a J‐point elevation of ≥0.1 mV above the baseline that was either notched (a positive J deflection at the QRS‐complex/ST‐segment transition) or slurred (a smooth transition from QRS to the ST segment) in at least 2 consecutive leads.^8^ Fragmented QRS was defined as ≥4 spikes in 1 or ≥8 spikes within the QRS complex in all of the leads (V1, V2, and V3).^6^ A prominent S wave in lead I was defined as S‐wave amplitude ≥0.1 mV and/or duration ≥40 ms.^14^ ECGs recorded before the initiation of pharmacological treatment were used for analysis. Measurements were performed blindly by 2 independent cardiologists (S.N. and N.K.). An ECG was recorded and analyzed using an electrocardiographic system with attached software (Fukuda Denshi Co, Tokyo, Japan) or scanned and imported to the computer and analyzed by the program developed by our institution. The authors had full access to the data and take full responsibility for their integrity.

### Electrophysiological Testing

After obtaining written informed consent from patients, an electrophysiological study was conducted in 90 patients, as described previously.^28^ The criterion for the induction of ventricular arrhythmia was VF or polymorphic ventricular tachycardia lasting >30 s or requiring direct current shock induced at a coupling interval of ≥200 ms with a maximum of 2 extrastimuli.

### Mutation Analysis of *SCN5A*

Genetic testing for mutations in the *SCN5A* gene was performed in 93 patients (44%), as previously described.^29^

### Statistical Analysis

Data were analyzed using JMP, version 13.0.0 (SAS, Cary, NC). Categorical variables were expressed as frequencies and percentages, and they were analyzed by the χ^2^ test. Continuous variables were expressed as the mean±SD. For continuous variables, comparisons between 2 unpaired groups were made using the Welch's *t* test. One‐way analysis of variance and the Tukey‐Kramer test were used for comparisons of continuous variables among the 3 groups. VF event was defined as the appropriate implantable cardioverter‐defibrillator shock against VF and aborted cardiac arrest attributable to VF. The receiver‐operating characteristic curve and the area under the curve were evaluated to select the optimized cutoff value for the prediction of VF during follow‐up. Survival curves were plotted by the Kaplan‐Meier method and analyzed by the log‐rank test. Event analysis over time was performed using the Cox proportional hazard regression model. Multivariate analysis estimated age, sex, history of syncope/VF, SCN5A mutation, early repolarization pattern, complete right bundle branch block, fragmented QRS, prominent S in lead I, and minimum late R′+S. Risk was quantified as a hazard ratio (HR) with a 95% confidence interval (CI). *P*<0.05 was considered statistically significant.

## Results

### Patient Characteristics

The baseline characteristics of the study population are summarized in Table 1. Age ranged from 16.0 to 78.3 years, with a median of 43.5 years. Two hundred patients (96%) were men, and 36 patients (17%) had a family history of sudden cardiac death. Twenty‐six patients (12%) had a history of VF episodes (group VF), 54 (26%) had a history of syncope episodes (group SY), and 129 (62%) did not have any symptoms (group AS). Gene analysis showed that an *SCN5A* gene mutation was present in 17 patients (18%). In the electrophysiological study, VF was induced in 22 patients (24%). An implantable cardioverter‐defibrillator was used in 79 patients. Table 2 represents the patient characteristics in each group (VF, SY, and AS). Age, sex, family history of sudden cardiac death, positive SCN5A mutation, and VF inducibility in the electrophysiological study did not differ among the groups.

**Table 1:** Clinical and ECG Characteristics in All Patients (n=209)

Characteristics	Values
Age, y	45±14
Male sex	200 (96)
Symptom	
Group VF	26 (12)
Group SY	54 (26)
Group AS	129 (62)
FH of SCD	36 (17)
Induced VF with PES (performed: n=90)	22 (24)
SCN5A mutation (performed: n=93)	17 (18)
ICD implantation	79 (38)
Spontaneous type 1 ECG	209 (100)
Inferolateral ER pattern	19 (9)
CRBBB	20 (10)
fQRS	44 (21)
Prominent S in lead I	111 (53)
No. of type 1 ECGs	4.5±2.1

Values are number (percentage) or mean±SD. AS indicates patients without any symptoms; CRBBB, complete right bundle branch block; ER, early repolarization; FH, family history; fQRS, fragmented QRS; ICD, implantable cardioverter‐defibrillator; No. of type 1 ECGs, the number of ECG leads showing type 1 morphological features among 9 right precordial leads; PES, programmed electrical stimulation; SCD, sudden cardiac death; SY, patients with a previous syncope episode; VF, (patients with documented) ventricular fibrillation.

**Table 2:** Clinical and ECG Characteristics in Each Group

Characteristics	Group VF	Group SY	Group AS	*P* Value
	(n=26)	(n=54)	(n=129)	
Age, y	41±12	45±13	45±14	NS
Male sex	26 (100)	50 (93)	124 (96)	NS
FH of SCD	5 (19)	12 (22)	19 (15)	NS
SCN5A mutation	3/18 (17)	9/35 (26)	5/40 (13)	NS
ICD implantation	26 (100)	36 (67)	17 (13)	<0.001
Induced VF with PES	6/15 (40)	9/39 (23)	7/36 (19)	NS
Inferolateral ER pattern	2 (8)	3 (6)	14 (11)	NS
CRBBB	7 (27)	5 (9)	8 (6)	<0.01
fQRS	11 (42)	13 (24)	20 (16)	<0.01
Prominent S in lead I	18 (69)	31 (57)	62 (48)	NS
No. of type 1 ECGs	5.1±2.1	4.6±2.5	4.3±2.0	NS

Values are number (percentage), number/total (percentage), or mean±SD. AS indicates patients without any symptoms; CRBBB, complete right bundle branch block; ER, early repolarization; FH, family history; fQRS, fragmented QRS; ICD, implantable cardioverter‐defibrillator; No. of type 1 ECGs, the number of ECG leads showing type 1 morphological features among 9 right precordial leads; NS, not significant; PES, programmed electrical stimulation; SCD, sudden cardiac death; SY, patients with a previous syncope episode; VF, (patients with documented) ventricular fibrillation.

### Baseline ECG Findings

**Figure 3:**
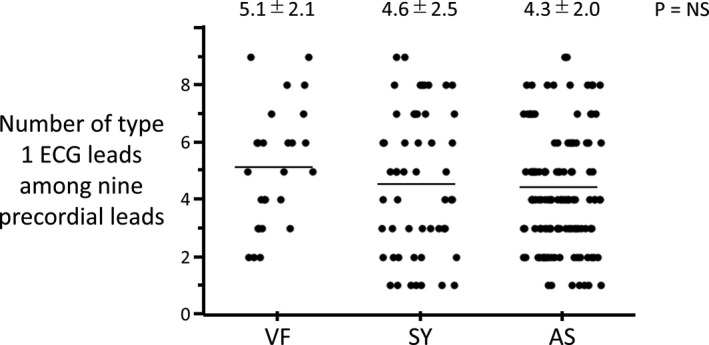
Distribution of the number of ECG leads showing type 1 in 3 groups. The number of ECG leads showing type 1 among the 9 right precordial leads did not differ among the 3 groups (*P*=not significant). The distribution points overlapped widely among the 3 groups. Numerical values show the mean±SD. AS indicates patient without any symptoms; SY, patient with a previous syncope episode; VF, patient with documented ventricular fibrillation.

The baseline ECG characteristics are summarized in Table 1. The inferolateral early repolarization pattern was observed in 19 patients (9%), and the number of ECG leads exhibiting type 1 morphological features was 4.5±2.1 (Table 1). The ECG characteristics in each group are summarized in Table 2. The number of ECG leads exhibiting type 1 morphological features among the 9 right precordial leads did not differ among the groups (Figure 3).

### Amplitude of Type 1 ECG

**Figure 4:**
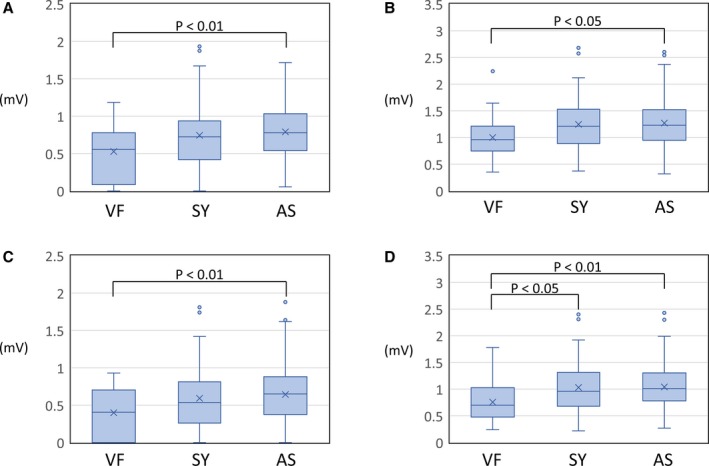
Box‐plot diagram showing the amplitude of type 1 ECG in each group. **A**, Averaged S‐wave amplitude of type 1 ECG. **B**, Averaged late R′ plus S‐wave amplitude of type 1 ECG. **C**, Minimum S‐wave amplitude of type 1 ECG. **D**, Minimum late R′ plus S‐wave amplitude of type 1 ECG. AS indicates patient without any symptoms; SY, patient with a previous syncope episode; VF, patient with documented ventricular fibrillation.

**Figure 5:**
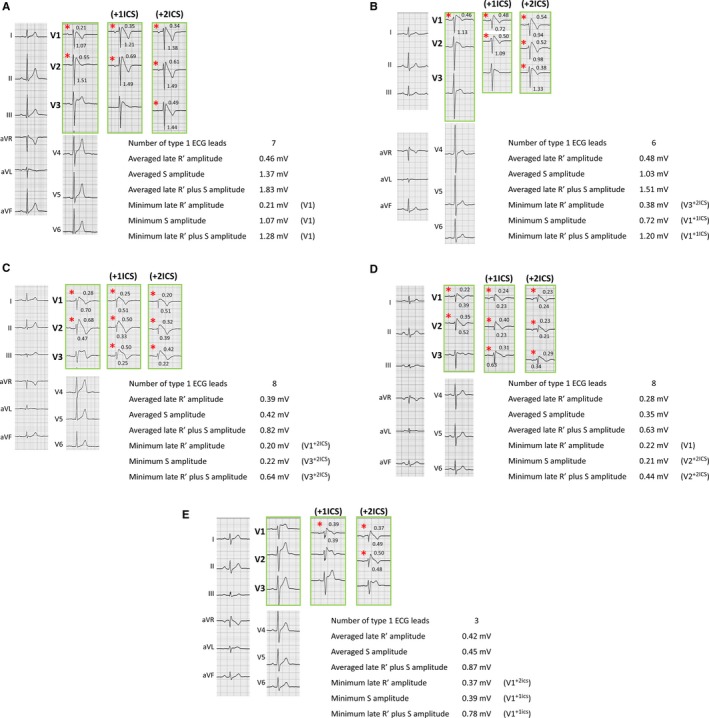
Representative cases. Representative cases of ECGs, including the 9 right precordial leads (green frame) and evaluated data in this study. Red asterisks indicate type 1 ECG. Numerical values on the ECG show the amplitude of later R′ (top panels) and S (bottom panels) in each ECG showing type 1. **A**, A 31‐year‐old male patient without any symptoms (group AS). This patient was free from all symptoms during follow‐up. Minimum late R′, minimum S, and minimum late R′ plus S were recorded in lead V1. **B**, A 66‐year‐old male patient with a previous syncope episode (group SY). This patient was free from all symptoms during follow‐up. Minimum late R′ was recorded in lead V3 with upper 2 intercostal spaces (+2ICS), and minimum S and minimum late R′ plus S were recorded in lead V1 with upper 1 intercostal space (+1ICS). **C**, A 52‐year‐old male group AS patient. In this patient, a ventricular fibrillation episode was observed during follow‐up. Minimum late R′ was recorded in lead V1^+2^ ^ICS^, and minimum S and minimum late R′ plus S were recorded in lead V3^+2^ ^ICS^. **D**, A 51‐year‐old male group SY patient. In this patient, a ventricular fibrillation episode was observed during follow‐up. Minimum late R′ was recorded in lead V1, and minimum S and minimum late R′ plus S were recorded in lead V2^+2^ ^ICS^. **E**, A 42‐year‐old male patients with documented ventricular fibrillation. In this patient, a recurrent ventricular fibrillation episode was observed during follow‐up. Minimum late R′ was recorded in lead V1^+2ICS^, and minimum S and minimum late R′ plus S were recorded in lead V1^+1ICS^.

Table 3 represents the amplitude of type 1 ECG in each group. Although the averaged and minimum amplitudes of the late R′ wave did not differ, the averaged and minimum amplitudes of the S wave and late R′ plus S wave differed among the 3 groups. The Tukey‐Kramer test revealed that the averaged S wave, averaged late R′ plus S wave, and minimum S wave were significantly smaller in the VF group than in the AS group, and the minimum late R′ plus S wave was significantly smaller in the VF group than in the SY and AS groups (Figure 4). Figure 5 shows representative ECG tracings and the evaluated data in this study.

**Table 3:** Amplitude of Type 1 ECGs in Each Group

Variable	Group VF	Group SY	Group AS	*P* Value
Averaged late R′, mV	0.47±0.23	0.50±0.19	0.48±0.19	NS
Averaged S, mV	0.52±0.38	0.74±0.45	0.79±0.38	<0.01
Averaged late R′ plus S, mV	1.00±0.40	1.25±0.49	1.27±0.44	<0.05
Minimum late R′, mV	0.30±0.14	0.38±0.16	0.34±0.14	NS
Minimum S, mV	0.40±0.32	0.59±0.44	0.64±0.37	<0.05
Minimum late R′ plus S, mV	0.76±0.36	1.03±0.48	1.04±0.40	<0.01

Values are mean±SD. AS indicates patients without any symptoms; NS, not significant; SY, patients with a previous syncope episode; VF, patients with documented ventricular fibrillation.

The median value of the minimum late R′ plus S wave was 0.97 mV. Table 4 presents the characteristics of patients in whom the minimum late R′ plus S wave was higher and lower than the median value. There was a statistically significant difference in the baseline clinical symptoms between the 2 groups that were higher and lower than the median value.

**Table 4:** Characteristics of Patients in Whom the Minimum Late R′ Plus S Wave Was Higher and Lower Than the Median Value

Characteristics	Minimum Late R′+S Wave		*P* Value
	Lower Than or Equal to the Median Value	Higher Than the Median Value	
	(n=105)	(n=104)	
Male sex	99 (94)	101 (97)	NS
Age, y	48±14	42±12	<0.001
Symptom			
Group VF	19 (18)	7 (7)	<0.05
Group SY	28 (27)	26 (25)	…
Group AS	58 (55)	71 (68)	…
FH of SCD	18 (17)	18 (17)	NS
SCN5A mutation	12/55 (22)	5/38 (13)	NS
Induced VF with PES	12/48 (25)	10/42 (24)	NS
ER pattern	7 (7)	12 (12)	NS
CRBBB	15 (14)	5 (5)	<0.05
fQRS	38 (36)	6 (6)	<0.001
Prominent S in lead I	62 (59)	49 (47)	NS
No. of type 1 ECGs	4.9±2.2	4.0±2.1	<0.01
VF event during follow‐up	24 (23)	5 (5)	<0.01

Values are number (percentage), number/total (percentage), or mean±SD. AS indicates patients without any symptoms; CRBBB, complete right bundle branch block; ER, early repolarization; FH, family history; fQRS, fragmented QRS; No. of type 1 ECGs, the number of ECG leads showing type 1 morphological features among 9 right precordial leads; NS, not significant; PES, programmed electrical stimulation; SCD, sudden cardiac death; SY, patients with a previous syncope episode; VF, (patients with documented) ventricular fibrillation.

### Arrhythmic Event and Predictor

**Figure 6:**
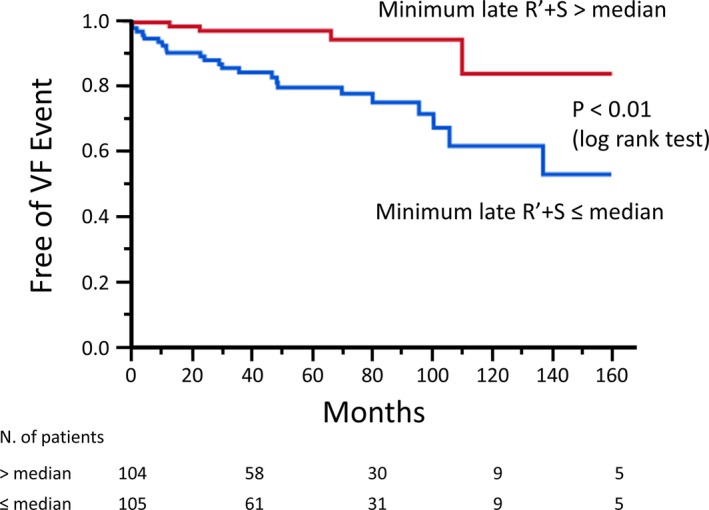
Kaplan‐Meier analysis of freedom from ventricular fibrillation (VF) event. Patients with the minimum late R′ plus S amplitude of type 1 ECG at or lower than the median value (0.97 mV) had a higher incidence of VF than others (log‐rank test, *P*<0.01).

During the follow‐up period of 56±48 months, VF events occurred in 29 patients (25 appropriate implantable cardioverter‐defibrillator shocks caused by VF and 4 aborted sudden cardiac deaths attributable to VF). Four patients died: 2 of them were suicides, 1 was lung cancer, and 1 was pneumonia. The date of death in each patient was the time when the patient actually died. These 4 patients were considered as right censored in analysis. Kaplan‐Meier analysis showed that the minimum amplitude of type 1 ECG at or lower than the median value was more significantly associated with the occurrence of VF (log‐rank test, *P*<0.01, Figure 6).

**Figure 7:**
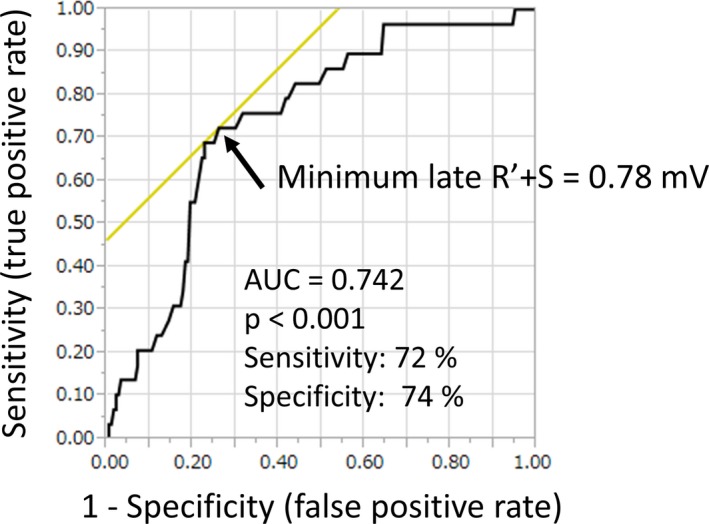
Receiver‐operating characteristic (ROC) curve and area under the curve (AUC). ROC curve comparing the sensitivity and specificity of the minimum late R′ plus S amplitude and future ventricular fibrillation (VF) event. ROC curve analysis for the amplitude of the late R′+ S wave in type 1 ECG revealed that the optimized cutoff point in the prediction of VF event during follow‐up was 0.78 mV.

Receiver‐operating characteristic curve analysis for the minimum late R′ plus S wave revealed that the optimized cutoff point in the occurrence of VF during follow‐up was 0.78 mV (area under the curve=0.742, *P*<0.001, Figure 7). For ease of measurement in the clinical setting, the cutoff value of the minimum late R′ plus S wave was approximated to 0.8 mV. The minimum late R′ plus S wave ≤0.8 mV had a sensitivity of 72%, a specificity of 73%, a negative predictive value of 94%, and a positive predictive value of 30%. Table 5 presents the characteristics of patients with a minimum late R′ plus S wave of ≤0.8 and >0.8 mV. Patients with a minimum late R′ plus S wave of ≤0.8 mV tended to have a higher age, a higher prevalence of complete right bundle branch block, and more ECG leads exhibiting type 1.

**Table 5:** Characteristics of Patients in Whom the Minimum Late R′ Plus S Wave Was Higher and Lower Than 0.8 mV

Characteristics	Minimum Late R′+S Wave		*P* Value
	≤0.8 mV	>0.8 mV	
	(n=69)	(n=140)	
Male sex	65 (94)	135 (96)	NS
Age, y	49±14	43±13	<0.01
Symptom			
Group VF	17 (25)	9 (6)	<0.001
Group SY	18 (26)	36 (26)	
Group AS	34 (49)	95 (68)	
FH of SCD	14 (20)	22 (16)	NS
SCN5A mutation	10/41 (24)	7/52 (13)	NS
Induced VF with PES	11/35 (31)	11/55 (20)	NS
ER pattern	6 (8)	13 (9)	NS
CRBBB	12 (17)	8 (6)	<0.01
fQRS	33 (48)	11 (8)	<0.001
Prominent S in lead I	41 (59)	70 (50)	NS
No. of type 1 ECGs	5.0±2.3	4.2±2.1	<0.05
VF event during follow‐up	21 (30)	8 (6)	<0.001

Values are number (percentage), number/total (percentage), or mean±SD. AS indicates patients without any symptoms; CRBBB, complete right bundle branch block; ER, early repolarization; FH, family history; fQRS, fragmented QRS; No. of type 1 ECGs, the number of ECG leads showing type 1 morphological features among 9 right precordial leads; NS, not significant; PES, programmed electrical stimulation; SCD, sudden cardiac death; SY, patients with a previous syncope episode; VF, (patients with documented) ventricular fibrillation.

Table 6 represents the univariate and multivariate analyses for the prediction of VF events during follow‐up. The prevalence of VF events was significantly higher in patients with previous VF episodes (HR, 5.329; 95% CI, 2.460–11.66; *P*<0.001), fragmented QRS (HR, 2.461; 95% CI, 1.147–5.171; *P*=0.021), and a minimum late R′ plus S wave of ≤0.8 mV (HR, 4.808; 95% CI, 2.202–11.591; *P*<0.001). Multivariate analysis revealed that previous syncope attack (HR, 14.764; 95% CI, 2.565–284.447; *P*=0.014), previous VF episode (HR, 29.607; 95% CI, 4.616–610.847; *P*=0.003), and a minimum late R′ plus S wave of ≤0.8 mV (HR, 4.154; 95% CI, 1.413–13.730; *P*=0.013) were independent risk factors for future VF events.

**Table 6:** Predictive Factors of VF Event

Factor	Univariate Analysis			Multivariate Analysis		
	HR	95% CI	*P* Value	HR	95% CI	*P* Value
Male/female ratio	0.814	0.169‐14.634	0.846	1.112	0.163‐22.197	0.926
Age	0.992	0.965‐1.020	0.606	0.999	0.960‐1.044	0.990
History of syncope	1.792	0.831‐3.745	0.133	14.764	2.565‐284.447	0.014
History of VF	5.329	2.460‐11.636	<0.001	29.607	4.616‐610.847	0.003
FH of SCD	0.980	0.327‐2.400	0.968	…	…	…
SCN5A mutation	2.482	0.987‐5.823	0.053	2.006	0.734‐5.183	0.157
Induced VF in PES	0.735	0.239‐1.886	0.541	…	…	…
ER pattern	1.742	0.511‐4.530	0.338	3.918	0.556‐17.389	0.102
CRBBB	1.793	0.661‐4.148	0.231	1.738	0.315‐8.201	0.493
fQRS	2.461	1.147‐5.171	0.021	0.846	0.240‐2.703	0.784
Prominent S in lead I	1.676	0.777‐3.899	0.191	0.588	0.198‐1.729	0.329
No. of type 1 ECGs	1.101	0.932‐1.301	0.254	…	…	…
Minimum late R′+S wave of ≤0.8 mV	4.808	2.202‐11.591	<0.001	4.154	1.413‐13.730	0.013

CI indicates confidence interval; CRBBB, complete right bundle branch block; ER, early repolarization; FH, family history; fQRS, fragmented QRS; HR, hazard ratio; No. of type 1 ECGs, the number of ECG leads showing type 1 morphological features among 9 right precordial leads; PES, programmed electrical stimulation; SCD, sudden cardiac death; VF, ventricular fibrillation.

**Figure 8:**
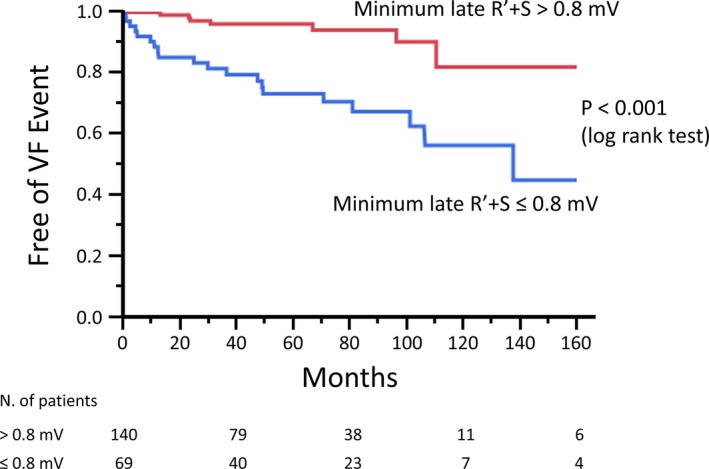
Kaplan‐Meier analysis of freedom from ventricular fibrillation (VF) event. Kaplan‐Meier analysis revealed that the patients with minimum late R′ plus S amplitude of ≤0.8 mV had a significantly worse prognosis.

Figure 8 shows the Kaplan‐Meier VF‐free survival curve in patients with a minimum late R′ plus S wave of ≤0.8 versus >0.8 mV. Patients with a minimum late R′ plus S wave of ≤0.8 mV had a significantly worse prognosis (*P*<0.001).

## Discussion

The present study showed that type 1 ECG with low amplitude of late R′ plus S waves on the right precordial lead positioned among the standard and upper 1 and 2 ICSs is highly and independently associated with fatal ventricular tachyarrhythmia in patients with BrS. The number of ECG leads exhibiting type 1 was not associated with fatal arrhythmic event.

Several articles reported that abnormal low‐voltage areas with fractionated potential were commonly observed on the RVOT epicardium in patients with BrS.^24^, ^25^, ^26^ Radiofrequency ablation of these substrates can eliminate the appearance of type 1 ECG and suppress the occurrence of VF events. Accordingly, the extent of abnormal epicardial low‐voltage area on the RVOT should be associated with the occurrence of VF.

The location of the RVOT has been reported to correlate with type 1 ECG recording on the right precordial leads.^22^, ^23^ Recording the right precordial leads on the standard and upper ICSs can result in the appearance of type 1 ECG with high sensitivity because the RVOT is commonly located below the right precordial lead on the standard and/or upper ICS. Moreover, because the right precordial leads use unipolar recording, they can reflect electrical information on the RVOT epicardium, although it is slightly apart. The usefulness of unipolar electrogram recordings, which can reflect the voltage of remote sites, has previously been reported.^30^

### Amplitude of Type 1 ECG

In general, the lower‐voltage areas associated with disease severity are arrhythmogenic substrates. Low‐voltage areas should contain abnormal myocardium with delayed and disrupted conduction that can lead to the development of reentrant tachyarrhythmia. We suspect that a low‐amplitude type 1 ECG can reflect low‐voltage areas on the RVOT epicardium and be associated with arrhythmogenesis. In this study, we found a significant correlation between low‐amplitude type 1 ECG and life‐threatening ventricular tachyarrhythmia in patients with BrS. Baseline data showed that the averaged S wave, averaged late R′ plus S wave, and minimum S wave were significantly smaller in the VF group than in the AS group and that the minimum late R′ plus S wave was significantly smaller in the VF group than in the AS and SY groups. Moreover, multivariate analysis revealed that the minimum amplitude of the late R′ plus S wave of ≤0.8 mV among the right precordial leads was an independent predictor of VF event during follow‐up. Several articles have reported the correlation between fatal arrhythmic event and ECG characteristics related to depolarization abnormality, such as an aVR sign, a fragmented QRS in the right precordial leads, prolonged QRS duration in the precordial leads, and wide and/or deep S wave in lead I.^5^, ^6^, ^9^, ^14^ Similarly, the results of our study underscored the importance of depolarization abnormality for the development of VF in BrS. The method evaluated in this study should also record the right precordial leads in higher ICSs. The measurement of amplitude is, however, uncomplicated compared with evaluation of the aVR sign, fragmented QRS, QRS duration, and S‐wave duration/depth.

1. The initial R wave in the right precordial leads and the initial Q wave in the left precordial leads commonly exhibit normal left‐to‐right depolarization of the interventricular septum, even in cases involving right bundle branch block.^31^, ^32^ Accordingly, we analyzed the amplitude of the late R′ wave and S wave but not the initial R wave in each ECG exhibiting type 1 in this study.
2. In this study, we analyzed the data without excluding the patients with complete right bundle branch block, because it is sometimes difficult to diagnose in those with BrS and would be inappropriate to exclude in risk assessment.

### Number of Type 1 ECG Leads

The results of this study indicated that the number of ECG leads exhibiting type 1 among the 9 right precordial leads was not associated with fatal arrhythmic event, although a low‐voltage type 1 ECG was predictive in patients with BrS. It has been reported that spontaneous type 1 ECG is one of the strong predictors for the occurrence of VF in BrS.^3^, ^10^, ^11^ Multiple ECG leads manifesting type 1 among the right precordial leads, including upper ICS recording, may demonstrate a broad substrate exhibiting type 1 morphological features on the surface ECG. We speculate that, although these type 1 morphological features are essential for the diagnosis of BrS, they are not sufficient for the occurrence and maintenance of VF in BrS. A past report also suggested that patients with BrS and a type 1 ECG recorded only on the upper ICS showed a similar prognosis as those with an ECG recorded on the standard ICS.^33^

### Mechanism of Type 1 ECG and VF Occurrence

The results of our study did not elucidate the mechanisms of ECGs exhibiting type 1 and the occurrence of polymorphic ventricular tachycardia and VF in BrS. A low‐voltage type 1 ECG can, however, correlate with low‐voltage areas on the RVOT epicardium and be critically associated with the development of fatal ventricular tachyarrhythmia. Correlation between the amplitude of type 1 ECG and epicardial voltage on the RVOT should be analyzed in future research.

### Study Limitations

This study has several limitations. First, it was a retrospective study conducted in a single center. The number of patients evaluated in this study was relatively modest. Prospective multicenter studies involving more patients are needed to confirm the results of our study. Second, circadian and daily fluctuations in the ECG were observed in those with BrS. In this study, we evaluated ECGs exhibiting type 1 with first documentation among the 9 right precordial leads. Repeated ECG recordings and their significance were not, however, examined. Third, considering the nature of BrS, a mean follow‐up of 56 months might be short. Finally, in the baseline data, we cannot deny the possibility that neurally mediated syncope was included in patients with syncope, because it is sometimes difficult to differentiate from truly arrhythmic syncope.

## Conclusions

In patients with BrS, a low‐voltage type 1 ECG among the right precordial leads on the standard and upper ICSs is highly and independently related to fatal ventricular arrhythmia.

## Disclosures

None.
